# Immunopathology of Airway Surface Liquid Dehydration Disease

**DOI:** 10.1155/2019/2180409

**Published:** 2019-07-14

**Authors:** Brandon W. Lewis, Sonika Patial, Yogesh Saini

**Affiliations:** Department of Comparative Biomedical Sciences, School of Veterinary Medicine, Louisiana State University, Baton Rouge, LA 70803, USA

## Abstract

The primary purpose of pulmonary ventilation is to supply oxygen (O_2_) for sustained aerobic respiration in multicellular organisms. However, a plethora of abiotic insults and airborne pathogens present in the environment are occasionally introduced into the airspaces during inhalation, which could be detrimental to the structural integrity and functioning of the respiratory system. Multiple layers of host defense act in concert to eliminate unwanted constituents from the airspaces. In particular, the mucociliary escalator provides an effective mechanism for the continuous removal of inhaled insults including pathogens. Defects in the functioning of the mucociliary escalator compromise the mucociliary clearance (MCC) of inhaled pathogens, which favors microbial lung infection. Defective MCC is often associated with airway mucoobstruction, increased occurrence of respiratory infections, and progressive decrease in lung function in mucoobstructive lung diseases including cystic fibrosis (CF). In this disease, a mutation in the *cystic fibrosis transmembrane conductance regulator* (*CFTR*) gene results in dehydration of the airway surface liquid (ASL) layer. Several mice models of *Cftr* mutation have been developed; however, none of these models recapitulate human CF-like mucoobstructive lung disease. As an alternative, the *Scnn1b* transgenic (*Scnn1b*-Tg+) mouse model overexpressing a transgene encoding *sodium channel nonvoltage-gated 1*, *beta subunit* (*Scnn1b*) in airway club cells is available. The *Scnn1b*-Tg+ mouse model exhibits airway surface liquid (ASL) dehydration, impaired MCC, increased mucus production, and early spontaneous pulmonary bacterial infections. High morbidity and mortality among mucoobstructive disease patients, high economic and health burden, and lack of scientific understanding of the progression of mucoobstruction warrants in-depth investigation of the cause of mucoobstruction in mucoobstructive disease models. In this review, we will summarize published literature on the *Scnn1b*-Tg+ mouse and analyze various unanswered questions on the initiation and progression of mucobstruction and bacterial infections.

## 1. Background

Aerobic processes within a cell consume oxygen (O_2_) and release carbon dioxide (CO_2_) during the process of respiration. Pulmonary ventilation is responsible for supplying O_2_ to and eliminating CO_2_ from cells undergoing aerobic respiration. In addition to oxygen, aberrant constituents of ambient air such as abiotic insults and airborne pathogens are also inhaled into the airspaces [1]. Upon entering the conducting airways, airborne insults are trapped within the airway surface liquid (ASL) layer, a thin layer of hydrated mucus that lines the airway epithelium. The airway epithelial cells are specialized to constitute a mucociliary clearance (MCC) host defense mechanism that facilitates the removal of trapped insults [1]. Ciliated cells move the layer of mucus containing the airborne insults towards the epiglottis, thus away from airspaces [1].

Defects in the functioning of the mucociliary escalator compromise the MCC of inhaled pathogens and abiotic insults, which favors airspace infection and lung injury, respectively [2]. Impaired MCC is also often associated with airway mucoobstruction in mucoobstructive lung disease patients [2]. The cause-effect relationship between these two responses and their effect on microbial infections are unclear. High morbidity and mortality among mucoobstructive disease patients, high economic and health burden, and lack of scientific understanding of the progression of mucoobstruction warrant in-depth investigation of the pathogenesis of mucoobstruction using mucoobstructive disease models [3–5]. In this review, we will focus our discussion on MCC defect in cystic fibrosis (CF) and its recapitulation in a widely accepted mouse model of CF, i.e., *Scnn1b*-Tg+ mouse.

## 2. Physiology of ASL Layer

**Figure 1:**
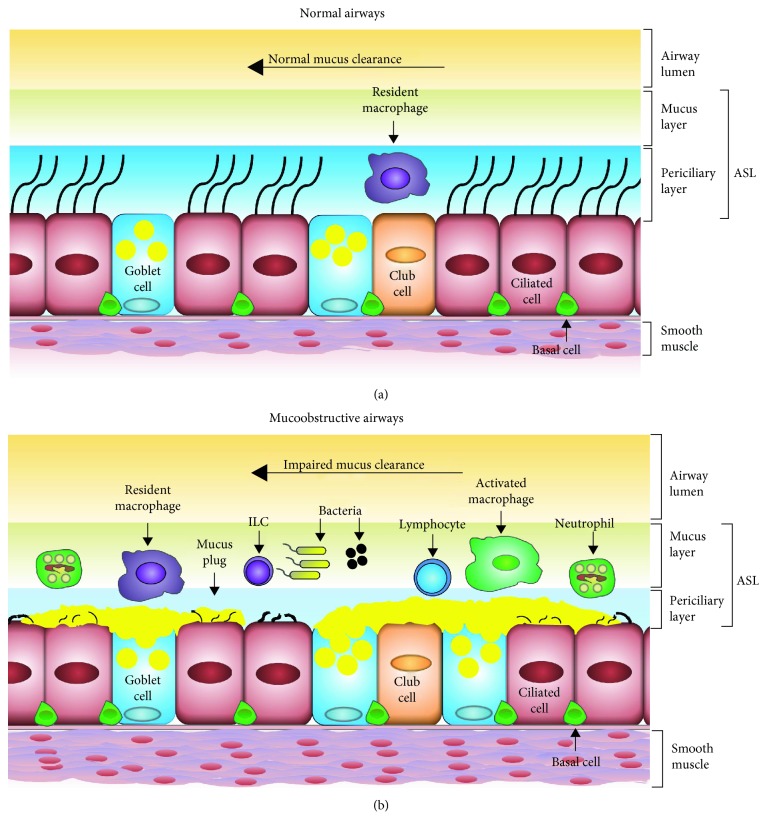
Diagrammatic comparisons of normal and mucoobstructive airways. (a) In normal airways, the normal functioning of epithelial ion channels maintains a healthy ASL layer. The normal functioning of the mucociliary clearance system efficiently clears aberrant ASL constituents. As a result, the epithelial layer consists of a balanced proportion of various epithelial cell types, including ciliated cells, club cells, and goblet cells. In addition, resident macrophages continue to perform their sentinel roles. (b) In mucoobstructive airways, an ion-channel defect causes ASL dehydration, which leads to mucus hyperconcentration, mucoobstruction, mucous cell metaplasia, bacterial infection, and airway inflammation.

**Figure 2:**
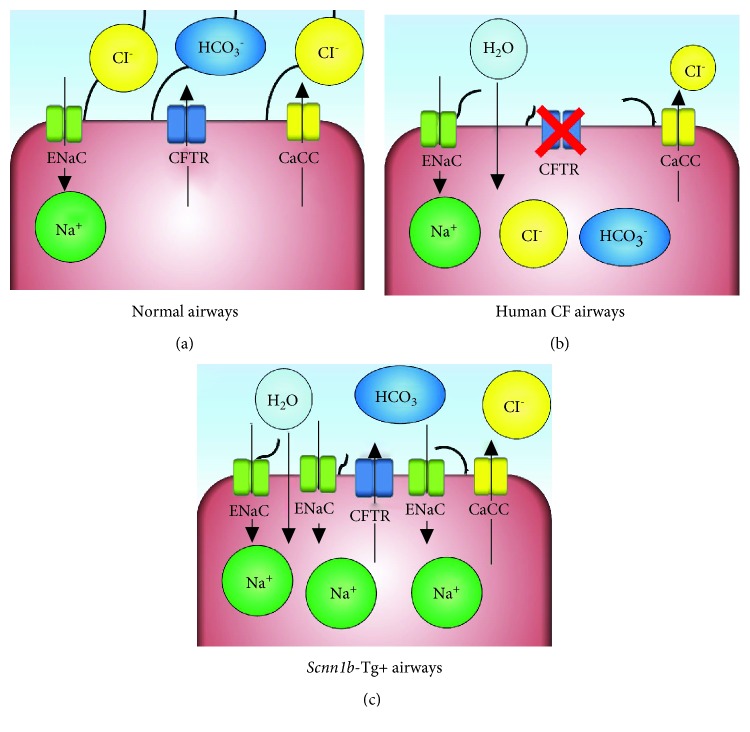
Ion-channel physiology in airways. (a) The hydration state of normal airways in airway surface liquid (ASL) is regulated by the concerted action of ion channels. Major ion channels responsible for regulating chloride (Cl^−^)/sodium (Na^+^) transport are the cystic fibrosis transmembrane conductance regulator (CFTR), calcium-activated chloride channels (CaCCs), and epithelial Na^+^ channels (ENaC). CFTR and CaCCs are responsible for regulating Cl^−^ transport, while ENaC facilitates epithelial Na^+^ absorption. CFTR is also responsible for bicarbonate (HCO_3_^−^) transport that regulates the local pH of the airways. Balanced ionic transport maintains water contents of ASL in the physiological range. (b) In cystic fibrosis, a dysfunctional CFTR channel results in the net movement of sodium ions into the cytoplasm of airway epithelial cells. The osmotic drive due to sodium hyperabsorption dictates the net movement of ASL water into the cytoplasm of airway epithelial cells. These alterations result in the pathology of CF lung disease. (c) In mice, the chloride ion transport inhibition due to the genetic inactivation of a CFTR channel defect is compensated by relatively more prominent CaCCs. In *Scnn1b*-Tg+ airways, the overexpression of ENaC results in the hyperabsorption of sodium ion into the cytoplasm of airway epithelial cells, an ionic imbalance defect similar to human CF airways.

The ASL layer, a thin layer of fluid that lines the luminal surface of the airway epithelium, is comprised of two distinct layers: the mucus layer and the periciliary layer [1]. The mucus layer is a luminal (superficial) layer of ASL that is exposed to the air and traps the airborne insults [6]. Removal of inhaled pathogens and abiotic insults involves unidirectional movement of the mucus layer towards the epiglottis [1]. Located directly underneath the mucus layer, the aqueous periciliary layer bathes the cilia projecting from the airway epithelium and facilitates ciliary beating [2]. The force generated by the ciliary beating within the periciliary layer fuels the movement of the mucus layer towards the epiglottis [1] (Figures 1(a) and 2(a)).

The two layers work on the gel-on-brush model in which the large membrane-tethered mucins and mucopolysaccharides of the periciliary layer form a brush-like network of polymers on the epithelial surface [6]. Electron microscopic examination of cultured human bronchial epithelial cells reveals the brush to be a meshwork consisting of large tethered macromolecules, i.e., MUC1, MUC4, MUC16, MUC20, and heparan sulfate, that are attached to the ciliary shaft and epithelial cell surface [6]. These large tethered macromolecules create a semipermeable gradient mesh that becomes tighter near the epithelial surface and is seemingly impenetrable to MUC5B, MUC5AC, and inhaled particles [6]. Button et al. determined that 2 nm dextran particles readily infiltrate the periciliary layer to reach the epithelial surface, while 40 nm particles are excluded from reaching the epithelial surface [6]. Thus, the brush acts as a size-exclusion barrier for infiltrating entities [6].

The periciliary brush also contributes to the regulation of ASL layer hydration by facilitating water distribution between the two layers [6]. Identical charges among the membrane-tethered macromolecules create intermolecular repulsive forces to create an osmotic pressure gradient that stabilizes the periciliary layer by opposing the osmotic pressure gradient created by the overlying mucus layer [6]. In healthy hydrated airways, osmotic pressure created by the brush keeps the mucus layer above the outstretched cilia and facilitates normal MCC [6]. During dehydration of the ASL layer in diseases such as CF, water is first drawn from the mucus layer, increasing the concentration of mucus and osmotic pressure [6]. As the pressure generated from the mucus layer increases, water is drawn from the mucus layer as well as the periciliary layer, resulting in ciliary compression and impaired MCC [6].

Contrary to a previous hypothesis that the periciliary layer is stationary, a study by Matsui et al. reveals that the periciliary layer is moved along with the mucus layer and dextran was cleared at a similar rate by both layers [7, 8]. Simple frictional interaction between the two layers does not account for the similar clearance rates [8]. Matsui et al. propose that a transfer of momentum takes place in order to facilitate the efficient movement of the two layers [8]. Ciliary beating promotes momentum transfer from the mucus layer to the periciliary layer, thus facilitating the concerted movement of both layers [8]. The transport rate and the contribution of both layers towards efficient MCC are determined by the amount and composition of the ASL.

The amount of ASL, expressed as the height of the ASL layer, is a critical factor for the normal functioning of the mucociliary escalator. While the height of the mucus layer varies depending on the airway location (7-70 *μ*m), the optimal height of the periciliary layer in human airways is approximately 7 *μ*m, approximately the height of outstretched cilia [1, 2, 9]. The height of the ASL layer is regulated by a concerted action of various ion channels on the apical surface of the airway epithelium [10]. Major ion channels responsible for regulating chloride (Cl^−^)/sodium (Na^+^) transport are the cystic fibrosis transmembrane conductance regulator (CFTR), calcium-activated chloride channels (CaCCs), and epithelial Na^+^ channels (ENaC) [10]. While epithelial excretion of Cl^−^ is regulated by CFTR and CaCCs, epithelial Na^+^ absorption is regulated by ENaC [10]. CFTR is also responsible for bicarbonate (HCO_3_^−^) transport that regulates the local pH of the airways [11]. The outcome of the concerted action of these ion channels regulates Cl^−^ and Na^+^ transport across the apical surface of airway epithelial cells, thus regulating the hydration status of the airway epithelium [10].

Another factor determining the efficient functioning of the MCC system is the percent solids in the ASL layer. The constituents of the ASL layer, including secreted mucins, immune cells, ions, antimicrobial peptides, and cytokines, account for approximately 2.5% of the solids in healthy airways [1, 12].

## 3. ASL Dehydration in CF: A Result of Single Ion-Channel Defect

CF lung disease exemplifies how the defective functioning of a single ion channel, i.e., CFTR, results in serious disturbances in ASL physiology (Figures 1(b) and 2(b)). With the loss of CFTR function in CF epithelial cells, Cl^−^ is retained within the epithelial cells while Na^+^ absorption by ENaC increases, leading to increased epithelial cytosolic NaCl contents [9, 13]. The increased cytosolic contents of NaCl in epithelial cells create an osmotic drive that promotes net movement of water from the ASL layer into the epithelial cells, thus leading to ASL layer dehydration.

The dehydration of the ASL layer results in the increased concentration of solutes (hyperconcentration) that leads to the compression of the periciliary layer by the overlying mucus layer, resulting in ciliary collapse and impaired MCC [6]. An increase from 2.5% to 6% solids, e.g., in CF airways, in the ASL layer compromises ciliary beat frequency and mucus layer transport [12]. Whether the increase in percent solids in mucoobstructive airways is a direct result of ASL layer dehydration or excessive accumulation or poor clearance of aberrant entities such as mucus plugs, microbes, inflammatory cells, and cellular debris, or a combination of all three outcomes, remains unclear.

## 4. Animal Models of ASL Dehydration

Although CF affects multiple organs, mucoobstructive lung disease is the major contributor to the morbidity and mortality associated with CF [14]. Various *Cftr*-knockout animal models including mice, pigs, ferrets, and rats have been generated with the intent of recapitulating mucociliary clearance impairment of human CF airways. In Sections 4.1, 4.2, 4.3, 4.4, and 4.5, the advantages and limitations of various animal models of impaired *Cftr* functioning and ASL dehydration will be discussed.

### 4.1. Mice

The availability of strains with genetic alterations of genes related to various inflammatory or pathological outcomes is an unmatched advantage of employing mouse as a disease model. Therefore, to recapitulate human CF-like lung disease, a number of mouse models have been developed over the past two decades (summarized in Table 1).

To begin with, in 1992, Snouwaert et al. generated the *Cftr^tm1UNC^* mouse via targeted disruption of the *Cftr* gene (Table 1) [15]. When compared with wild-type (WT) mice, *Cftr^tm1UNC^* mice exhibited mortality due to intestinal mucoobstruction; however, contrary to many pathological changes observed in human CF patients, these mice did not exhibit significant pathological changes in the pancreas, male reproductive system, liver, and gallbladder [15]. Although mucoobstruction and bacterial infection were not observed in the airways in *Cftr^tm1UNC^* mice, an increase in goblet cells in the proximal airways and impaired MCC were observed [15, 16]. Although the *Cftr^tm1UNC^* mouse model exhibited impaired MCC upon bacterial challenge, it did not recapitulate the spontaneously arising airway mucoobstruction and bacterial infection observed in CF patients.

In 1992, Dorin et al. generated the *Cftr^tm1HGU^* mouse model also via targeted disruption of exon 10 [17]. Similar to *Cftr^tm1UNC^* mice, the *Cftr^tm1HGU^* mice exhibited no pathological abnormalities in the pancreas and reproductive system, although one male exhibited increased mucus accumulation in the vas deferens [17]. Unlike the *Cftr^tm1UNC^* mouse model, however, the *Cftr^tm1HGU^* mouse model exhibited only mild intestinal mucoobstruction and all pups were able to survive past weaning [17]. Although the airway mucoobstructive phenotype associated with CF was not observed, upon challenge with two types of bacteria commonly associated with CF, i.e., *Staphylococcus aureus* and *Burkholderia cepacia*, *Cftr^tm1HGU^* mice exhibited pathological features of CF lung disease [18]. *Cftr^tm1HGU^* mice exhibited difficulty in clearing the bacteria from the airspaces as effectively as WT littermates [18]. The airways of *Cftr^tm1HGU^* mice also exhibited a marked increase in the abundance of goblet cells and mucoobstruction in response to bacterial challenge [18]. Although the *Cftr^tm1HGU^* mouse model, similar to *Cftr^tm1UNC^*, exhibited impaired MCC in response to challenge, it also did not exhibit the spontaneously occurring mucoobstructive phenotype seen in CF airways.

Similar to the previously mentioned models, Ratcliff et al. targeted exon 10 of *Cftr* to generate the *Cftr^tm1CAM^* mouse model [19]. Similar to *Cftr^tm1UNC^*, *Cftr^tm1CAM^* pups exhibited increased mortality attributed to intestinal mucoobstruction [19]. Similar to human CF patients, *Cftr^tm1CAM^* mice exhibited obstruction of the pancreatic ducts, a phenotype not observed in *Cftr^tm1UNC^* and *Cftr^tm1HGU^* mice [19]. An interesting phenotype observed in the *Cftr^tm1CAM^* model that was previously not reported in *Cftr^−/−^* mice was the susceptibility to ocular infections and lacrimal gland abnormalities [19]. Although the *Cftr^tm1CAM^* mouse exhibited increased mortality, intestinal mucus obstruction, and pancreatic abnormalities, this model still did not exhibit mucus accumulation in the airways as seen in CF [19].

In 1993, O'Neal et al. generated the *Cftr^tm1BAY^* mouse model by targeted disruption of exon 3 in the *Cftr* locus [20]. No pathological abnormalities, i.e., mucus obstruction, were observed in the lungs of *Cftr^tm1BAY^* [20]. This mouse model also exhibited increased mortality-associated muco-obstruction of the intestines [20]. In 1995, Hasty et al. targeted exon 2 of the *Cftr* in order to generate the *Cftr^tm3BAY^* mouse model [21]. In accordance to the previous mouse models, *Cftr^tm3BAY^* mice exhibited high mortality as a result of severe intestinal mucoobstruction [21]. *Cftr^tm3BAY^* mice also did not exhibit an onset of lung disease, liver disease, or obstruction of the pancreatic ducts when examined at birth, one week of age, or three to four weeks of age [21]. A clinical phenotype that is commonly associated with male CF patients is sterility [15]. Contrary to the phenotype observed in male CF patients, *Cftr^tm3BAY^* males exhibited no reproductive abnormalities, whereas most females were sterile [21]. In 1996, Rozmahel et al. generated the *Cftr^tm1HSC^* mouse model on a 129/SV background by targeted disruption of exon 1 [22]. As seen in previously discussed models, the *Cftr^tm1HSC^* mouse model exhibited severe intestinal mucoobstruction that led to high mortality rates [22].

In 1995, two mouse models, i.e., *Cftr^tm2CAM^* and *Cftr^tm1KTH^*, incorporating a deletion of phenylalanine at position 508 (ΔF508) of the *Cftr* gene locus, the most common genetic mutation associated with human CF, were generated by two separate groups [23, 24]. Although significant mortality due to intestinal mucoobstruction was observed in both strains, no abnormalities in the pancreas, male reproductive system, or lungs were evident [23, 24]. In 1995, a third mouse, *Cftr^tm1EUR^*, with the ΔF508 mutation was generated on an FVB background [25]. As seen in *Cftr^tm2CAM^* and *Cftr^tm1KTH^*, pathological abnormalities, i.e., mucus retention, were not observed in the lungs, pancreas, liver, or vas deferens in these mice. However, these mice did not exhibit mortality due to intestinal mucus obstruction, but they did exhibit hypertrophy of goblet cells in the intestines [25]. The observed differences in the rate of mortality may be attributed to the strain background.

Also in 1996, Delaney et al. generated a mouse model possessing another mutation associated with human CF, the G551D mutation (*Cftr^G551D^*) that occurs in approximately 3% of CF patients [26]. The *Cftr^G551D^* also exhibited increased mortality due to intestinal mucoobstruction [26]. No pathological differences in the lungs, pancreas, and reproductive system were observed in *Cftr^G551D^* mice [26]. In 2002, Dickinson et al. generated the *Cftr^tm2HGU^* targeted integration of the G480C mutation, a mutation associated with human CF [27]. The *Cftr^tm2HGU^* exhibited comparable survival to WT littermates, and no intestinal mucoobstruction was observed [27]. Mild goblet cell hypertrophy was observed in the intestines of the *Cftr^tm2HGU^* mice [27]. There were no abnormalities reported in the lungs and the reproductive systems [27]. The *Cftr^tm2UTH^* model was generated by the integration of the R117H mutation, a mutation characterized by CFTR reaching the apical surface of the epithelium but not properly functioning [28]. Upon challenge with *Pseudomonas aeruginosa*, the *Cftr^tm2UTH^* mouse model exhibited significantly lower neutrophil counts as compared to similar inflammatory responses to a previous *Cft*r^−/−^ mouse model, i.e., *Cftr^tm1UNC^*, but presented no significant differences in inflammatory cytokine levels [28]. The *Cftr^tm3UTH^* mouse model was generated by integrating the Y122X mutation [28]. The *Cftr^tm3UTH^* mouse model exhibited lower levels of tumor necrosis factor alpha (TNF-*α*) and - interleukin 1 beta (IL-1*β*) when compared to the *Cftr^tm1UNC^* mouse model in response to *Pseudomonas aeruginosa* [28]. Taken together, there were no substantial differences between the tested *Cftr^−/−^* mouse models in response to *Pseudomonas aeruginosa* challenge [28]. Although mouse models of *Cftr* knockdown or various functional mutations recapitulated the intestinal mucoobstruction, none of these models produced the spontaneous onset of airway mucoobstruction and airway bacterial infection exhibited in CF, warranting a need for an animal model that recapitulates human CF.

### 4.2. Pig

In order to address the limitations observed in CF mouse models, Rogers et al. generated *Cftr*^−/−^ pigs that exhibited gastrointestinal, pancreatic, and reproductive abnormalities commonly associated with CF [29]. All *Cftr^−/−^* piglets exhibited meconium ileus, a phenotype seen in ~15% of human CF patients [29]. The pancreas of *Cftr^−/−^* piglets was morphologically smaller when compared with WT littermates and exhibited ductal obstruction [29]. Male *Cftr^−/−^* pigs were also infertile, a phenotype commonly associated with human male CF patients [29]. Pertaining to lung disease manifestation, no lung inflammation, mucus obstruction, or infection was observed at 6-12 hours after birth [29, 30]. However, *Cftr^−/−^* newborn pigs exhibited difficulty in clearing bacteria upon challenge, i.e., *Staphylococcus aureus* [30]. *Cftr^−/−^* pigs that survived more than two months exhibited delayed onset of lung disease characterized by airway inflammation and mucoobstruction [30]. Although the *Cftr^−/−^* pig phenotypically expressed common hallmarks of CF, the mucoobstructive airway phenotypes in this model have been described as variable, ranging from no to severe manifestation [30].

### 4.3. Ferret

In 2010, Sun et al. generated the *Cftr^−/−^* ferret by targeted disruption that exhibited meconium ileus, pancreatic lesions, degenerate or absent vas deferens, dehydration of the ASL layer, severe airway inflammation, and a predisposition to lung infections [31]. Due to the susceptibility to lung infections, antibiotic treatment was necessary for the survival of the *Cftr^−/−^* ferret [32]. The *Cftr^−/−^* ferret exhibited mortality by the age of six months with antibiotic treatment, with 3 of 11 *Cftr^−/−^* ferrets surviving [32]. In order to investigate the progression of lung disease, Sun et al. removed the *Cftr^−/−^* ferret from antibiotics at three months of age [32]. Upon cessation of antibiotic treatment, progressive lung disease that resembled human CF, i.e., mucoobstruction and bacterial colonization, was observed in the major and small airways [32]. Thus, antibiotic intervention was needed to enhance survival to induce a more applicable CF lung phenotype in the *Cftr*^−/−^ ferret.

### 4.4. Rat

In 2014, Tuggle et al. generated the *Cftr^−/−^* rat model by targeted disruption [33]. There was no significant difference in survival between *Cftr^−/−^* and WT littermates until weaning but survival was drastically decreased in the *Cftr^−/−^* rat by the age of six weeks [33]. Decreased survival was a result of intestinal mucoobstruction and complications [33]. There were no pancreatic abnormalities observed in the *Cftr^−/−^* rat [33]. Although the ASL layer in the *Cftr^−/−^* rat model was dehydrated, no pathological abnormalities were observed in the lungs at the age of 22 to 42 days [33]. Abnormal lung pathology developed as the *Cftr^−/−^* rats aged due to the development of submucosal gland hypertrophy [34]. At six months of age, the small airways of the *Cftr^−/−^* rat exhibited increased mucus secretion and accumulation leading to delayed mucus transport [34]. The *Cftr^−/−^* rat model did not exhibit airway obstruction or spontaneously arising bacterial infection [34].

While nonmurine *Cftr*^−/−^ models have been somewhat successful in recapitulating the human CF-like spontaneous mucoobstruction and bacterial infection, unlike mice models, it remains challenging to introduce genetic changes into their genomes. The *Scnn1b*-Tg+ mouse model, although with an intact *Cftr* gene, exhibits CF-like lung pathology. In Section 4.5, we will review the characteristics of this strain and modulation of *Scnn1b*-Tg+ lung disease upon various other genetic alterations.

### 4.5. *Scnn1b*-Tg+ Mouse Model

None of the *Cftr*^−/−^ mice models spontaneously recapitulate human CF-like disease, most likely due to the functional compensation by CaCCs [35]. To circumvent this issue, the *Scnn1b*-Tg+ mouse was generated to accomplish increased Na^+^ absorption into the airway epithelial cells [36]. The increased Na^+^ absorption in *Scnn1b*-Tg+ mice was achieved via overexpressing a transgene encoding *sodium channel nonvoltage-gated 1*, *beta subunit* (*Scnn1b*) in club cells (Figure 2(c)) [36]. Na^+^ absorption is enhanced in tracheal tissues of adult and neonatal *Scnn1b*-Tg+ mice; the Cl^−^ secretion remained unaffected (Figure 2(c)) [36].

These mice exhibit various features of mucoobstructive airway diseases. The increased Na^+^ absorption into the airway epithelium of *Scnn1b*-Tg+ mice is evident as early as postnatal day (PND) 3 that results in the dehydration of the ASL layer leading to mucoobstruction and impaired MCC [36, 37]. A longitudinal study revealed that high mortality (~50% in the first two weeks of life) is a result of asphyxiation related to airway mucoobstruction [37]. The *Scnn1b*-Tg+ mice exhibited difficulty in clearing bacteria upon challenge with *Haemophilus influenzae* and *Pseudomonas aeruginosa* [36].

The initial microbiological studies on bronchoalveolar lavage fluid (BALF) from *Scnn1b*-Tg+ adult mice failed to detect spontaneous bacterial infection [36]. Since the initial microbiological studies were conducted only in adult mice and speculating that mucoobstruction creates a microaerophilic environment, Livraghi-Butrico et al. hypothesized that *Scnn1b*-Tg+ mice would be more susceptible to pulmonary infections by microaerophilic bacteria in neonatal age when the immune system is underdeveloped as compared to adult *Scnn1b*-Tg+ mice [38]. Under microaerophilic conditions, BALF from *Scnn1b*-Tg+ neonates showed the presence of polymicrobial bacterial species of oropharyngeal origin [38].

The *Scnn1b*-Tg+ mouse model also exhibited necrosis of epithelial cells in the airways at newborn (PND 0.5) and neonatal (PND 3.5) stages [37]. Interestingly, epithelial cell hypoxia was observed in the mucoobstructive airways of *Scnn1b*-Tg+ mice [37]. It is likely that the hypoxic stress to the airway epithelial cells caused by mucoobstruction leads to epithelial necrosis [37]. The blood gas analyses on neonatal (PND 3.5-5.5) *Scnn1b*-Tg+ mice revealed a significant reduction in the partial pressure of oxygen (*P*_O2_) and oxygen saturation, indicative of a systemic hypoxic environment [37]. This is most likely a result of bronchopulmonary dysplasia or emphysematous changes that are evident in *Scnn1b*-Tg+ mice. The *Scnn1b*-Tg+ mice manifest airway inflammation accompanied by granulocyte (neutrophil and eosinophil) infiltration and macrophage activation [37].

In the remaining parts of this review, we will discuss various immune cells in the context of muco-obstructive disease evolution in *Scnn1b*-Tg+ mice.

## 5. Macrophages

Macrophages are key sentinel cells that express pro- or anti-inflammatory functions based on the external cytokine milieu, broadly classified as M1 and M2 activation, respectively. M1 macrophages are associated with the elimination of pathogens and the secretion of proinflammatory cytokines, e.g., IL-1, IL-6, and IL-23 [39]. M1 macrophages also facilitate the expansion of TH17 lymphocytes that recruit neutrophils through the secretion of IL-17 [39]. Stimulation of M1 macrophages is facilitated by interferon gamma (IFN-*γ*), lipopolysaccharide (LPS), and other activators of Toll-like receptors (TLRs) [40–42]. Most of the TLRs require an adaptor molecule, myeloid differentiation factor 88 (MyD88), to initiate a downstream intracellular signaling cascade [43]. The MyD88 pathway leads to the activation of nuclear factor-kappa B (NF-*κ*B), a key transcription factor in M1 activation that regulates the expression of a variety of inflammatory genes, e.g., *TNF-α*, *IL1β*, and *interleukin* 6 (*IL-6*) [44].

M2 macrophages are associated with parasitic infection, tissue remodeling, and promotion of Th2 responses [44]. Stimulation of M2 macrophages is facilitated through IL-4, IL-13, and IL-10 [45–47]. IL-4 and IL-13 facilitate the polarization of M2 macrophages through signal transducer and activator of transcription (STAT) 6, whereas IL-10 acts through STAT3 [48, 49].

Mall et al. initially observed morphological activation of pulmonary macrophages at two weeks, a phenotype that was found to persist into adulthood [37]. To profile molecular signatures of macrophages as they relate to the development of mucoobstructive lung disease, we performed gene expression analyses on purified *Scnn1b*-Tg+ macrophages at four disease-relevant time-points, i.e., PND 0 (less than 24 hours of age), 3, 10, and 42 [50]. There was evidence of both M1 and M2 macrophages in the BALF of *Scnn1b*-Tg+ mice at PND 3, with M1 as the more robust polarization state [50]. The predominance of M1 macrophages at PND 3 was found to be consistent with the presence of pulmonary bacterial infection typical of *Scnn1b-*Tg+ neonates [50]. The macrophage activation status experienced a shift to the M2 state at PND 10, and M2 was found to be more robust at PND 42 [50]. The robust molecular signatures exhibited by pulmonary macrophages during the progression of mucoobstructive lung disease in *Scnn1b*-Tg+ mice indicated their critical role in disease pathogenesis [50].

To elucidate the role of pulmonary macrophages in neonatal (PND 5-7) *Scnn1b*-Tg+ mice, we generated *Scnn1b*-Tg+ mice with macrophage deficiency [51]. In this strain, the expression of apoptosis-inducing diphtheria toxin A (DTA) was targeted to pulmonary macrophages via the myeloid cell-specific Lysozyme M (LysM) promoter [51, 52]. The superimposition of impaired MCC on macrophage depletion (DTA^+^-*Scnn1b*-Tg+) resulted in ~51% mortality due to an emaciated phenotype characterized by reduced weight gain, “flaky discoloration,” lethargy, and mortality [51]. Interestingly, macrophage depletion affected various inflammatory characteristics, i.e., alveolar space consolidation, airway inflammation, mucoobstruction, immune cell infiltration, and bacterial infection in *Scnn1b*-Tg+ [51]. The macrophage-depleted *Scnn1b*-Tg+ mice exhibited a significantly higher bacterial burden [51]. Although there was a presence of additional bacterial species, *Pasteurella pneumotropica* remained the predominant microbial inhabitant in the airways of macrophage-depleted mice.

To elucidate the contribution of pulmonary macrophages in mucoobstructive lung disease in adulthood, we compared the lung pathology of surviving macrophage-depleted adult mice [53]. Adult mice with macrophage deficiency exhibited a significantly higher degree of alveolar space consolidation [53]. Interestingly, DTA^+^-*Scnn1b*-Tg+ adult mice exhibited a significantly higher degree of mucoobstruction in airways and an increased number of mucus-producing cells compared to DTA^−^-*Scnn1b*-Tg+ littermates [53]. Taken together, these mechanistic reports that focused on the numerical depletion of macrophages highlighted the critical roles of these cells in the pathogenesis of lung disease in *Scnn1b-Tg+* mice.

*Matrix metalloproteinase*- (*MMP*-) *12*, a candidate genetic contributor to the development of emphysema, was found to be upregulated in the lungs of *Scnn1b*-Tg+ mice [54]. MMP12 was also found to be significantly upregulated in BALF macrophages of CF patients [54]. Trojanek et al. determined that MMP12 proteolytic activity was significantly higher on the surface of activated BALF macrophages of *Scnn1b*-Tg+ mice [54]. The administration of pharmacological inhibitors as well as the genetic deletion of *Mmp12* in *Scnn1b*-Tg+ mice significantly reduced mean linear intercepts and destructive index [54]. Since MMP12 is expressed in non-macrophage cells as well, it remains to be determined whether inactivation or deletion of macrophage-originated *Mmp12* accounts for the amelioration of alveolar space pathology in *Scnn1b*-Tg+ mice. Further investigation employing macrophage-specific deletion of various functionally relevant genes is necessary to determine the effect of functionally compromised macrophages on various pathological features of *Scnn1b-Tg+* mice.

## 6. Neutrophils

Neutrophils are cells of the innate immune system that are typically the first cells to be recruited during inflammation and serve to eliminate invading pathogens [55]. Neutrophils employ a variety of mechanisms for bacterial killing, e.g., phagocytosis, degranulation, or release of neutrophil extracellular traps (NETs) [56]. In the process of phagocytosis, neutrophils engulf pathogens that are subsequently encapsulated in phagosomes [55]. Encapsulated pathogens are killed by the use of NADPH oxygenase-dependent mechanisms (reactive oxygen species) or antibacterial proteins contained within the neutrophilic granules [55]. These neutrophilic granules can also be released extracellularly through the process of degranulation in order to act upon extracellular pathogens [55]. Highly activated neutrophils produce NETs that can immobilize the pathogens for subsequent phagocytosis or directly kill the entrapped pathogens [56]. NETs are also composed of antimicrobial proteins and enzymes, e.g., lactoferrin, cathepsin, and neutrophil elastase (NE), responsible for the elimination of invading pathogens [56]. Interestingly, NE has been linked to both beneficial and detrimental roles in the pathogenesis of CF [57].

The S*cnn1b*-Tg+ mice exhibited neutrophilic airspace infiltration accompanied by increased expression of neutrophil chemoattractants, i.e., keratinocyte chemoattractant (KC), lipopolysaccharide-induced CXC chemokine (LIX), macrophage inflammatory protein 2 (MIP-2), and granulocyte-colony-stimulating factor (G-CSF), beginning in the neonatal stages and persisting into adulthood [37, 38]. NE has been implicated in the induction of emphysema [58], mucous cell metaplasia (MCM), and mucus hypersecretion [59, 60]. The ablation of *Ne* in *Scnn1b*-Tg+ mice resulted in a significant decrease in lung volume, mean linear intercepts, and destructive index as compared to *Scnn1b*-Tg+ littermates [57]. The *Ne^−/−^*-*Scnn1b*-Tg+ mice also had reduced MCM and expression levels of genes associated with goblet cells and mucus secretion, i.e., *Gob5*, *Muc5ac*, and *Muc5b*, involved in this response [57]. These results suggested that compromised neutrophil function via NE deletion ameliorates lung pathology in *Scnn1b*-Tg+ mice [57].

Myeloid differentiation primary response 88 (MyD88) is a cytosolic adaptor molecule that is required for the downstream signaling upon TLR ligation. The ablation of the *Myd88* gene in *Scnn1b*-Tg+ mice resulted in significantly increased mortality when compared to *Myd88^+/-^-Scnn1b*-Tg+ littermates [38]. *Myd88^−/−^-Scnn1b*-Tg+ mice also exhibited significantly increased bacterial burden by a greater diversity of bacterial species [61]. As compared to *Myd88^+/-^-Scnn1b*-Tg+ mice, the *Myd88^−/−^-Scnn1b*-Tg+ mice exhibited a significant reduction in neutrophils and BALF levels of neutrophil chemokines, i.e., KC, LIX, MIP-2, and G-CSF [38]. These data suggested that the ablation of TLR signaling in *Scnn1b*-Tg+ mice leads to the reduced production of neutrophil chemoattractants and poor neutrophil recruitment; thus, there is poor bacterial clearance.

## 7. Eosinophils

Eosinophils are granulated cells of the innate immune system that respond to helminths and allergies [62]. The eosinophilic granules have been found to contain IL-4, IL-6, IL-10, and TNF-*α* [63]. As compared to WT mice, the *Scnn1b*-Tg+ mice exhibited significantly increased eosinophilia that, unlike neutrophilia that persisted into adulthood, peaked during the juvenile (2-3 weeks) stages and subsided during the adult stages [37]. The eosinophil chemoattractant, Eotaxin 1, was found to be overexpressed in the *Scnn1b*-Tg+ mouse as compared to WT littermates [37]. The ablation of *interleukin-* (*IL-*) *4 receptor alpha* (*Il4rα*), the receptor for IL-4 and IL-13, significantly reduced eosinophilic infiltration in 10-day old *Scnn1b*-Tg+ mice, suggesting the involvement of IL4R*α* ligands in eosinophilic recruitment [64, 65]. However, the exact role of eosinophils in *Scnn1b*-Tg+ lung disease is not yet clear.

## 8. Natural Killer Cells

Natural killer (NK) cells are cells of the innate immune system that are responsible for eliminating tumor cells and virally infected cells [66, 67]. Johannson et al. found that NK cells determine “self” from “nonself” through the recognition of major histocompatibility complex class I (MHC-I) [68]. NK cells not only possess the ability to kill target cells but also possess the ability to produce IFN-*γ* and TNF-*α* [69]. Through the production of IFN-*γ*, NK cells have also been shown to be involved in the differentiation of Th1 lymphocytes [70]. While the levels of IFN-*γ* and TNF-*α* are found to be elevated in the BALF from *Scnn1b*-Tg+ mice, whether NK cells are involved in the pathogenesis of *Scnn1b*-Tg+ mice remains unexplored.

## 9. T-Lymphocytes

The cells of the adaptive immune system, i.e., T- and B-lymphocytes, possess antigen-specific surface receptors that undergo recombination in order to mature [71]. The recombination is facilitated through *recombinase activating gene*- (*RAG*-) *1* and *RAG-2* [72]. T-lymphocytes are cells of the adaptive immune system and can functionally be divided into subsets, e.g., Th1, Th2, Th17, and T regulatory lymphocytes (Tregs) [73].

Th1 lymphocytes are responsible for controlling intracellular pathogens and are associated with the production of TNF-*α* and interferon-gamma (IFN-*γ*) [73]. Th2 lymphocytes are associated with the production with IL-4, IL-5, and IL-13 [73, 74]. As discussed previously, IL-4 and IL-13 have been linked to MCM and increased mucus production [75, 76]. Th17 lymphocytes secrete IL-17, a key proinflammatory cytokine associated with neutrophil recruitment [77]. Th17 lymphocytes are present during the early stages of CF, and a significant correlation exists between IL-17 and the total number of neutrophils [78]. *Pseudomonas aeruginosa* infection in BALF from CF patients is associated with significantly higher Th17-associated cytokines (IL-17, IL-6, IL-1*β*, and IL-8) [79]. Tregs are associated with the suppression of exacerbated Th2/Th17 inflammation [80]. CF patients with chronic *Pseudomonas aeruginosa* infection exhibited lower Treg counts when compared to CF patients without *Pseudomonas aeruginosa* infection [81].

Lymphocyte counts tend to be higher in BALF from *Scnn1b*-Tg+ mice as compared to their WT littermates, but a significant increase is evident only in adult *Scnn1b*-Tg+ mice [37]. While IFN-*γ* remains comparable between *Scnn1b*-Tg+ and their WT littermates, BALF levels of TNF-*α*, a Th1-associated cytokine, are elevated in the BALF of *Scnn1b*-Tg+ neonates [37]. Mall et al. found significantly higher levels of IL-13 starting at one week of age and waning after three weeks of age [37]. During this time, the *Scnn1b*-Tg+ mouse model exhibited significantly increased MCM and mucoobstruction [37]. A detailed analysis of the lungs for the presence of various subtypes of Th cells is warranted to completely understand the Th-associated responses in the *Scnn1b*-Tg+ mice.

## 10. B-Lymphocytes

B-lymphocytes (B-cells) secrete antigen-specific immunoglobulins (Ig) that constitute antigen-specific humoral immunity. In addition to bone marrow, spleen, and lymph nodes, B-cells localize in the tertiary lymphoid structures such as bronchus-associated lymphoid tissue (BALT) that surrounds bronchi in the lungs. BALTs are frequently found in CF patients and *Scnn1b*-Tg+ adult mice [38, 53, 82].

CF patients exhibit significantly higher levels of *Pseudomonas*-specific IgG antibodies [83]. Secretory IgA levels are also significantly upregulated in the nasal secretions of CF patients infected with *Pseudomonas aeruginosa* [84]. Livraghi-Butrico et al. found that *Myd88^−/−^-Scnn1b*-Tg+ mice exhibited significantly more lymphoid aggregates at eight weeks of age than *Myd88^+/-^-Scnn1b*-Tg+ littermates [38]. In our recent report, a significant increase in the presence of BALTs in the lung parenchyma of macrophage-deficient *Scnn1b*-Tg+ adults was observed [53]. The presence of these lymphoid aggregates was associated with higher levels of immunoglobulin (Ig) subtypes, i.e., IgA, IgM, IgG1, IgG2b, and IgG3, in BALF from macrophage-deficient *Scnn1b*-Tg+ adults [53]. While the antigen specificity of these immunoglobulins remains to be investigated, their increased levels in mice with BALTs likely reflect local adaptive response to bacterial infections.

## 11. Innate Lymphoid Cells (ILCs)

Innate lymphoid cells (ILCs) are innate immune cells that secrete Th effector cytokines but lack antigen-specific receptors that require recombination [85]. In simpler description, the ILCs (ILC1, 2, and 3) are the amnestic equivalent of Th subtypes (Th1, Th2, and Th17) [85]. ILCs delineate separately from T lymphocytes based on the expression of the transcription factor inhibitor of DNA binding 2 (Id2) [86]. ILCs also require the common cytokine receptor *γ*-chain (also known as Il2rg) [87]. ILCs have been classified based on their expression of transcription factors and cytokines [87].

ILC1s differentiate independently from NK cells from Id2 expressing common helper ILC precursors (ChILPs) [86]. Moro et al. identified ILC2s in mouse mesentery that produce high levels of Th2-associated cytokines, i.e., IL-5 and IL-13, in response to IL-33 [88]. Subsequent studies revealed that ILC2s could also produce the Th2-associated cytokine IL-4 and rely on GATA-3 for differentiation and maintenance [89, 90]. Takatori et al. identified ILC3s that produce Th17-associated cytokines, i.e., IL-17 and IL-22, in response to IL-1*β* as well as IL-23 and produce IL-17 and IL-22 [91]. ILC3s rely on the expression of rare orphan receptor- (ROR-) *γ*t for differentiation [87]. The role of ILCs in the pathogenesis and progression of lung disease in the *Scnn1b*-Tg+ mouse model remains unclear and warrants extensive investigation.

Given the predominance of Th-mediated responses in *Scnn1b-Tg+* mice of different ages, it is critical to characterize ILC as well as Th populations in the *Scnn1b-Tg+* mice. These studies when followed by ILCs and Th subtype depletion studies will dissect cell-specific roles in mucoobstructive lung disease in *Scnn1b-Tg+* mice.

## 12. Spontaneous Bacterial Infection in CF

CF is characterized by early bacterial colonization by microbes originating from the oral cavity and progressively shifts to a pathogen-dominated environment [92]. Muhlebach et al. conducted a longitudinal study to characterize the microbiome in young CF patients [92]. The lower airways of CF infants were determined to be relatively sterile, but microbes commonly associated with the oral cavity, e.g., *Streptococcus* and *Prevotella*, were predominant in the airways by the age of two years [92]. Of note, the origin of spontaneous bacterial colonization in *Scnn1b*-Tg+ neonates was also determined to be oropharyngeal [38]. At four years of age, the microbiome analyses from CF patients revealed the presence of pathogenic bacterial species, e.g., *Staphylococcus aureus*, *Haemophilus influenzae*, and *Pseudomonas aeruginosa* [92]. The presence of a pathogenic species in CF patients was associated with significantly increased inflammation and structural damage in the lungs [92]. Coburn et al. found that CF patients over the age of 25 exhibited a prevalence of *Pseudomonas aeruginosa* that was associated with declining lung function [93]. The progressive decline in lung function associated with *Pseudomonas aeruginosa* infection leads to respiratory failure and death in CF patients [94].

## 13. Does Infection Lead to the Airway Inflammation?

Whether inflammatory responses in mucoobstructive airways originate from infectious agents remained unclear until recently. To determine if the bacterial infection is essential for airway inflammation in *Scnn1b*-Tg+ mice, Livraghi-Butrico et al. rederived *Scnn1b*-Tg+ mice in a germ-free environment [38]. While, as expected, the germ-free *Scnn1b*-Tg+ mice did not exhibit airway bacterial colonization, other phenotypes including airway inflammation, macrophage activation, MCM, and airway mucoobstruction were still present [38]. Indeed, the macrophage activation was found to be more exaggerated in germ-free *Scnn1b*-Tg+ mice as compared to specific pathogen-free *Scnn1b*-Tg+ mice [50]. These results suggested that the inflammatory responses observed in germ-free *Scnn1b*-Tg+ mice were not dependent on the presence of microbes or pathogen-associated molecular patterns (PAMPs). Along the same lines, antibiotic treatment of spontaneously-infected *Cftr*^−/−^ ferrets failed to mitigate airway inflammation [95]. Therefore, it is likely that the ASL dehydration-induced stress to the airway cells, i.e., epithelium and immune cells, induces the release of proinflammatory damage-associated molecular patterns (DAMPs) that, in turn, mediates inflammatory responses in the airways.

Various DAMPs have been implicated in the pathogenesis of mucoinflammatory outcomes including airway inflammation, mucin hypersecretion, and MCM. IL-1*α*, a potent inducer of neutrophilic recruitment, is present at significantly higher levels in BALF from 5-day-old *Scnn1b*-Tg+ pups [96, 97]. The genetic deletion of *Il1r1*, a gene encoding the receptor for IL-1*α* and IL-1*β*, abolishes airway neutrophilia and significantly reduces mortality, mucoobstruction, and emphysema in *Scnn1b*-Tg+ pups [97]. Another DAMP, high-mobility group box 1 (HMGB1), is elevated in the sputum from CF patients [98]. Interestingly, HMGB1 levels are also elevated in the BALF from *Scnn1b*-Tg+ mice [98]. Since HMGB1 acts as a ligand for TLR2 and TLR4, its effect is expected to produce responses similar to PAMPs (LPS and lipoteichoic acid) [99, 100].

IL-33, a potent stimulator of Th2-associated responses, acts as a potent DAMP upon release by airway epithelial cells into the airspaces [101]. IL-33 binds to the ST2 receptor that is present on mast cells, macrophages, Th2 cells, and type 2 innate lymphoid cells (ILC2s) [102]. Administration of IL-33 induces the production of cytokines by Th2 lymphocytes *in vivo* [103]. IL-33 has also been linked to the activation of ILC2s that also release Th2-associated cytokines [104]. IL-33 levels are elevated in the juvenile *Scnn1b*-Tg+ mice [105]. Secondhand-smoke exposure to *Scnn1b*-Tg+ mice results in diminished IL-33 expression and BALF levels, which is strongly associated with diminished MCM and reduced expression of MCM-associated genes [105]. Further investigation on the mice with a genetic deletion of IL-33 on an *Scnn1b*-Tg+ background will confirm the role of IL-33 in the manifestation of mucoobstructive responses.

## 14. Does Mucous Cell Metaplasia (MCM) Lead to Mucoobstruction?

MCM refers to an epithelial remodeling response that increases the number of mucous cells in the airway epithelium and upregulates the expression of genes involved in mucin expression and secretion. *Scnn1b*-Tg+ mice exhibit a significantly higher number of mucous cells in proximal and distal airways as compared to their WT littermates [37]. Interestingly, the neonatal (PND 3.5) *Scnn1b*-Tg+ pups exhibit mucoobstruction in the trachea but in the absence of MCM, suggesting that mucus accumulation, rather than mucus overproduction, contributes to mucus plugging at this early age [37]. However, in 2−3-week-old *Scnn1b-*Tg+ mice, mucoobstruction along with MCM is found to be most prominent in the large and distal conducting airways, a feature that persisted into the adult *Scnn1b*-Tg+ mice [37, 105].

MCM is commonly associated with Th2-associated cytokines, i.e., IL-4 and IL-13 [75, 76]. As discussed before, the ablation of *Il4rα*, a common receptor for IL-4 and IL-13, significantly decreases neonatal mortality, MCM, and eosinophilic inflammation in the 10-day-old *Scnn1b*-Tg+ mice [65]. Interestingly, the ablation of *Il4rα* does not alter the severity of mucus plugging [65]. It appears that the normal production rate of mucus in the ASL-dehydrated state is capable of producing mucoobstruction; however, further experiments are required to ascertain this possibility.

## 15. Conclusions

Due to the high morbidity and mortality associated with CF-like mucoobstructive lung disease, an in-depth investigation of the immunological responses initiated as a result of ASL dehydration and mucoobstruction is warranted. Although several mice models incorporating different *Cftr* mutations are available, none of the mouse models effectively recapitulate CF-like mucoobstructive lung disease. Although not modulating the functioning of CFTR channels, the *Scnn1b*-Tg+ mouse model effectively demonstrates how a single ion-channel defect results in an imbalance in ion transport, which ultimately leads to ASL dehydration and associated lung disease.

The earliest manifestation of lung disease, i.e., ASL layer dehydration, mucoobstruction, immune cell infiltration, and spontaneous bacterial infections, exhibited in the *Scnn1b*-Tg+ mouse model provides a most representative model for the investigation of the pathogenesis and progression of human CF-like lung disease. The *Scnn1b*-Tg+ mouse also presents an outstanding tool to investigate the impact of various environmental insults, e.g., cigarette smoke, nanoparticles, and fungal spores, on the development and progression of mucoobstructive lung disease [105–107].

A complete understanding of the evolution of various pathological manifestations in this strain is still unclear. The availability of numerous genetic strains on a congenic C57BL/6 background presents an opportunity to investigate the development of complex mucoobstructive lung disease which otherwise is challenging to pursue. A list of studies employing various genetic alterations has been summarized in Table 2. Selective introduction of additional genetic alterations into the *Scnn1b*-Tg+ strain have already begun to dissect the pathway-specific roles of various genes in the pathogenesis of mucoobstructive lung disease.

## Figures and Tables

**Table 1:** Mouse models of single ion-channel defect.

S. No.	Model name	Strain background	Transgene/mutation	Spontaneous onset of lung disease	Airway mucus obstruction	Airway mucous cell metaplasia	Spontaneous airway bacterial infection	Chronic airway inflammation	Mortality	Reference
*1*	*Cftr^tm^ 1UNC*	C57BL/6	Mutation (exon 10)	No	Absent	Present only upon bacterial challenge	Absent	Neutrophilic infiltration at day 30	Yes	(Snouwaert et al., [15])
*2*	*Cftr^tm^ 1HGU*	MF1	Mutation (exon 10)	No	Absent	Present only upon bacterial challenge	Absent	Absent	No	(Dorin et al., [17])
*3*	*Cftr^tm^ 1CAM*	C57BL/6	Mutation (exon 10)	No	Absent	Absent	Absent	Absent	Yes	(Ratcliff et al., [19])
*4*	*Cftr^tm^ 1BAY*	C57BL/6 × 129	Mutation (exon 3)	No	Absent	Absent	Absent	Absent	Yes	(O'Neal et al., [20])
*5*	*Cftr^tm^ 3BAY*	129/Sv	Mutation (exon 2)	No	Absent	Absent	Absent	Absent	Yes	(Hasty et al., [21])
*6*	*Cftr^tm^ 2CAM*	C57BL/6	Mutation (*ΔF508*)	No	Absent	Absent	Absent	Absent	Yes	(Colledge et al., [23])
*7*	*Cftr^tm^ 1EUR*	FVB	Mutation (*ΔF508*)	No	Absent	Absent	Absent	Absent	No	(van Doorninck et al., [25])
*8*	*Cftr^tm^ 1KTH*	C57BL/6 × 129	Mutation (*ΔF508*)	No	Absent	Absent	Absent	Absent	Yes	(Zeiher et al., [24])
*9*	*Cftr^tm^ 1HSC*	129/SV	Mutation (exon 1)	No	Absent	Absent	Absent	Absent	Yes	(Rozmahel et al., [22])
*10*	*Cftr^G5^ 51D*	CD1/129	Mutation (*G551D*)	No	Absent	Absent	Absent	Absent	Yes	(Delaney et al., [26])
*11*	*Cftr^tm^ 2HGU*	C57BL/6 × 129	Mutation (*G480C*)	No	Absent	Absent	Absent	Absent	No	(Dickinson et al., [27])
*12*	*Scnn1b*-Tg+	C3H : C57	Transgene (*Scnn1b*)	Yes	Yes	Yes	Postnatal	Yes	Yes	(Mall et al., [36])
*13*	*Cftr^tm^ 2UTH*	C57BL/6	Mutation (*R117H*)	No	Absent	Absent	Absent	Absent	No	(van Heeckeren et al., [28])
*14*	*Cftr^tm^ 3UTH*	C57BL/6	Mutation (*Y122X*)	No	Absent	Absent	Absent	Absent	Yes	(van Heeckeren et al., [28])

**Table 2:** Various genetic modifications in the *Scnn1b*-Tg+ mouse model.

Genotype	Description	Macrophage infiltration	Neutrophil infiltration	Eosinophil infiltration	Lymphocyte infiltration	Mucous cell metaplasia	Airway mucus obstruction	Distal airspace enlargement	Reference
*Tnfα^−/−^*-*Scnn1b*-Tg+	Global deletion of *Tnfα*	No significant difference	No significant difference	No significant difference	No significant difference	No significant difference	No significant difference	No significant difference	(Livraghi et al., [65])
*Tnfr1^−/−^*-*Scnn1b*-Tg+	Global deletion of *Tnfr1*	No significant difference	No significant difference	No significant difference	No significant difference	No significant difference	No significant difference	No significant difference	(Livraghi et al., [65])
*Il4rα^−/−^*-*Scnn1b*-Tg+	Global deletion of *Il4rα*	No significant difference	No significant difference	Significantly reduced at PND 10 and 5 weeks of age	No significant difference	Significantly reduced at PND 10	No significant difference	No significant difference	(Livraghi et al., [65])
*Myd88^−/−^Scnn1b*-Tg+	Global deletion of *Myd88*	Significantly higher at PND 10; no significant difference at other observed time-points	Significantly reduced	No significant difference	Lymphoid hyperplasia significantly increased at 8 weeks of age	Significantly reduced at PND 5-7, but not at other time-points	Significantly reduced at PND 5-7, but not at any other observed time-point	Not reported	(Livraghi-Butrico et al., [38])
*Ne^−/−^-Scnn1b*-Tg+	Global deletion of *neutrophil elastase*	No significant difference	Significantly reduced	No significant difference	No significant difference	Significantly reduced	No significant difference	Significantly reduced	(Gehrig et al., [57])
*Il1r^−/−^*-*Scnn1b*-Tg+	Global deletion of *Il1r*	No significant difference	Significantly reduced	No significant difference	No significant difference	Not reported	Significantly reduced	Significantly reduced	(Fritzsching et al., [97])
DTA^+^-*Scnn1b*-Tg+	Partial deficiency of macrophages	No significant difference in total number of macrophage infiltration, significantly reduced in total percentage	Significantly increased	No significant difference	Increased occurrence of lymphoid aggregates in adult mice; significant infiltration in nonemaciated phenotype	Significantly reduced in emaciated phenotype	Significantly reduced in emaciated phenotype	No significant difference	(Saini et al., [51, 53])
*Muc5b^−/−^-Scnn1b*-Tg+	Global deletion of *Muc45b*	No significant difference	No significant difference	Not reported	Increased lymphoid aggregates, but no significant difference in BALF lymphocytes	Not reported	Significantly reduced	No significant difference	(Livraghi-Butrico et al., [108])
*Muc5ac^−/−^-Scnn1b*-Tg+	Global deletion of *Muc5ac*	No significant difference	No significant difference	Not reported	No significant difference in incidence of lymphoid aggregates	Not reported	Significantly reduced	Not reported	(Livraghi-Butrico et al., [108])
*Spdef^−/−^*-*Scnn1b*-Tg+	Global deletion of *Spdef*	No significant difference	Significantly increased in neonates	No significant difference	No significant difference	Not reported	No significant difference	Not reported	(Chen et al., [109])
